# A CMR study assessing aortic valve hemodynamics post-transcatheter aortic valve implantation compared to surgical aortic valve replacement

**DOI:** 10.1186/1532-429X-14-S1-P96

**Published:** 2012-02-01

**Authors:** Timothy Fairbairn, Christopher D Steadman, Adam N Mather, Manish Motwani, Daniel Blackman, Sven Plein, Gerry P McCann, John P Greenwood

**Affiliations:** 1 Multidisciplinary cardiovascular research centre, University of Leeds, Manchester, UK; 2 Cardiovascular sciences, University of Leicester, Leicester, UK; 3 Cardiology, Leeds General Infirmary, Leeds, UK

## Summary

Transcatheter aortic valve implantation (TAVI) removes the valvular impedance associated with aortic stenosis but can be complicated by significant aortic regurgitation. Using cardiovascular magnetic resonance we assessed the change in trans-aortic pressure gradient and regurgitant fraction 6 months post-TAVI and compared this to a surgical aortic valve replacement (SAVR) cohort. TAVI was superior at reducing aortic pressure gradient and equal to SAVR at reducing aortic regurgitation at 6 months.

## Background

Transcatheter aortic valve implantation (TAVI) is a rapidly developing procedure for the treatment of patients with severe aortic stenosis (AS) at high surgical risk. A reduction in trans-aortic pressure gradient is important to allow the ventricle to reverse remodel, but post-operative aortic regurgitation is a commonly reported complication. The quantification of aortic regurgitation (AR) by echocardiography and fluoroscopic techniques is limited, in part due to the paravalvular nature of regurgitation. We aimed to use cardiovascular magnetic resonance (CMR) imaging to determine the change in trans-aortic pressure gradient and accurately quantify paravalvular and valvular regurgitation post-TAVI compared to surgical aortic valve replacement (SAVR).

## Methods

**Figure 1:**
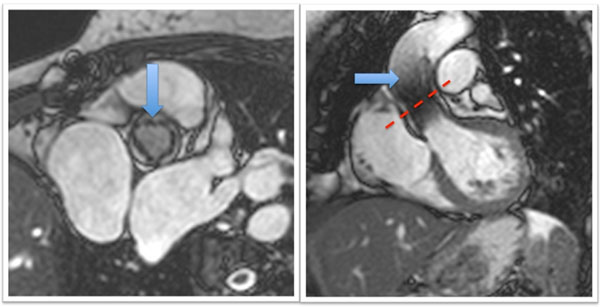
Short axis and coronal views of the CoreValve™ prosthesis (blue arrow). The typical scanning position for phase contrast imaging is represented by the red dashed line.

Fifty high-risk patients (EuroSCORE ≥20) with severe AS (peak velocity >4m/s) underwent TAVI (n=25) or SAVR (n=25). TAVI was performed using the Medtronic CoreValve prosthesis (26mm (20%) and 29mm (80%). Bioprosthetic SAVR of varying sizes (18-26mm) were used in 24 (96%) and mechanical 1 (4%) patients. Baseline and 6 month post-operative scans were performed on 1.5T systems (Phillips Intera or Siemens Avanto) using identical protocols. Through-plane velocity encoded (VENC) phase contrast imaging was performed perpendicular to the aortic valve jet at the aortic sinotubular junction (Figure 1, VENC 250-500cm/s, retrospective gating, slice thickness 6mm, 40 phases, FOV 340mm). Aortic flow was quantified using QFlow (Medis, Netherlands) to provide a peak forward flow velocity (m/s), forward flow volume (ml), backward flow volume (ml) and calculation of regurgitant fraction (%).

## Results

AS severity was similar between the TAVI and SAVR groups (50±16 vs. 55±20mmHg, P=0.7 respectively). Post-operatively the trans-aortic pressure gradient was significantly lower in both groups (TAVI 21±8mmHg, P<0.001; SAVR 35±13mmHg, P<0.001), although when compared to SAVR the TAVI group had a significantly greater reduction in their pressure gradient (P=0.017). Aortic regurgitant fraction at baseline was similar between the TAVI and SAVR groups (16±11 vs. 18±7%, P=0.4 respectively). Valve replacement/implantation resulted in an 8% reduction of AR in both groups, reaching statistical significance in the TAVI group (P=0.003) but not in the SAVR group (P=0.09). However, comparison of the procedures by 2-way ANOVA showed no difference (P=0.46) in their efficacy to reduce AR.

## Conclusions

TAVI results in a greater improvement in aortic valve pressure gradient compared to SAVR and reduces aortic regurgitation to a similar degree. This is clinically important as a lower aortic pressure gradient may encourage greater left ventricular (LV) reverse remodeling and post-operative aortic regurgitation may result in pulmonary oedema and impaired ventricular remodeling.

## Funding

None.

